# Detailed Characterization of Sympathetic Chain Ganglia (SChG) Neurons Supplying the Skin of the Porcine Hindlimb

**DOI:** 10.3390/ijms18071463

**Published:** 2017-07-07

**Authors:** Anna Kozłowska, Anita Mikołajczyk, Mariusz Majewski

**Affiliations:** 1 Department of Human Physiology, Faculty of Medical Sciences, University of Warmia and Mazury Olsztyn, Olsztyn 10-082, Poland; Mariusz.Majewski@uwm.edu.pl; 2 Department of Public Health, Epidemiology and Microbiology, Faculty of Medical Sciences, University of Warmia and Mazury Olsztyn, Olsztyn 10-082, Poland; asm@uwm.edu.pl

**Keywords:** skin-projecting neurons, chemical coding, sympathetic chain ganglia, pig

## Abstract

It is generally known that in the skin sympathetic fibers innervate various dermal structures, including sweat glands, blood vessels, arrectores pilorum muscles and hair follicles. However, there is a lack of data about the distribution and chemical phenotyping of the sympathetic chain ganglia (SChG) neurons projecting to the skin of the pig, a model that is physiologically and anatomically very representative for humans. Thus, the present study was designed to establish the origin of the sympathetic fibers supplying the porcine skin of the hind leg, and the pattern(s) of putative co-incidence of dopamine-β-hydroxylase (DβH) with pituitary adenylate cyclase-activating polypeptide (PACAP), somatostatin (SOM), neuronal nitric oxide synthase, substance P, vasoactive intestinal peptide, neuropeptide Y (NPY), leu5-enkephalin and galanin (GAL) using combined retrograde tracing and double-labeling immunohistochemistry. The Fast Blue-positive neurons were found in the L_2_–S_2_ ganglia. Most of them were small-sized and contained DβH with PACAP, SOM, NPY or GAL. The findings of the present study provide a detailed description of the distribution and chemical coding of the SChG neurons projecting to the skin of the porcine hind leg. Such data may be the basis for further studies concerning the plasticity of these ganglia under experimental or pathological conditions.

## 1. Introduction

It is generally known that skin is mainly innervated by sympathetic nerve fibers, with an additional complement of autonomic nerve fibers. In the human, autonomic nerve fibers derive from two sources: sympathetic and, rarely, parasympathetic neurons [1]. The sympathetic innervations of the skin was characterized in humans [2,3,4] and other mammals, such as the rat [5], guinea pig [6], cat [7], rabbit [8], dog [9] and pig [4]. These fibers innervate dermis, blood and lymphatic vessels, arteriovenous anastomoses, erector pili muscles, apocrine and eccrine glands, as well as hair follicles [10]. However, they are involved principally in the regulation of blood flow by two distinct branches: a noradrenergic vasoconstrictor branch and a cholinergic active vasodilator branch [11]. Noradrenergic vasoconstrictor nerves are tonically active in normothermic environments [12] and they increase activity during cold exposure, secreting norepinephrine, which acts on postsynaptic α_1_- and α_2_-receptors [13,14,15] to decrease skin blood flow [16]. Several studies have also shown that sympathetic innervations of the skin, in addition to their basic function in the blood flow, also plays an important role in the mechanisms of radicular and neuropathic pain [17,18,19,20].

In humans and cats, skin-projecting sympathetic neurons supplying the lower leg/hind paw were distributed in a distinct set of sympathetic chain ganglia (SChG). For instance, in humans such neurons were found in L_1_–L_4_ SChG [21], whereas in cats they were found within L_6_–S_2_ ganglia [22].

Previous studies in the pig showed that the majority of the postganglionic SChG neurons projecting to different organs, i.e., ovary [23], colon [24,25], urinary bladder [26,27] or pylorus [28] are noradrenergic, i.e., they express enzymes participating in the synthesis of noradrenaline—tyrosine hydroxylase (TH) and dopamine β-hydroxylase (DβH). Furthermore, there is strong evidence indicating that postganglionic sympathetic perikarya, in addition to classic catecholaminergic transmitter (noradrenaline), could contain other biologically active substances, such as: substance (SP), galanin (GAL), neuropeptide Y (NPY), neuronal nitric oxide synthase (nNOS), somatostatin (SOM), leu5-enkephalin (LENK), vasoactive intestinal peptide (VIP) or pituitary adenylate cyclase-activating polypeptide (PACAP) [24,25,26,27,28,29]. It was also strongly suggested that some of these markers co-operate with noradrenaline in cutaneous active vasoconstriction [30] while others participate in active vasodilation of cutaneous arterioles [31]. However, until now, little has been known about the distribution and chemical coding of sympathetic skin-projecting neurons supplying the porcine hind leg. Such data could be of great importance because the pig shows distinct anatomical, physiological, biochemical and immunological similarities to humans [32], which makes it a more suitable model than rodents [33] for studying human skin diseases [34]. Therefore, the present experiment was aimed at: (1) identifying the distribution pattern of retrogradely-labeled sympathetic SChG neurons involved in skin innervations of porcine hindlimb; and (2) elucidating the chemical phenotypes of noradrenergic SChG neurons using double-immunofluorescence.

## 2. Results

### 2.1. The Number and Distribution Pattern of Skin-Projecting Neurons with in the Porcine Sympathetic Chain Ganglia (SChG)

**Figure 1:**
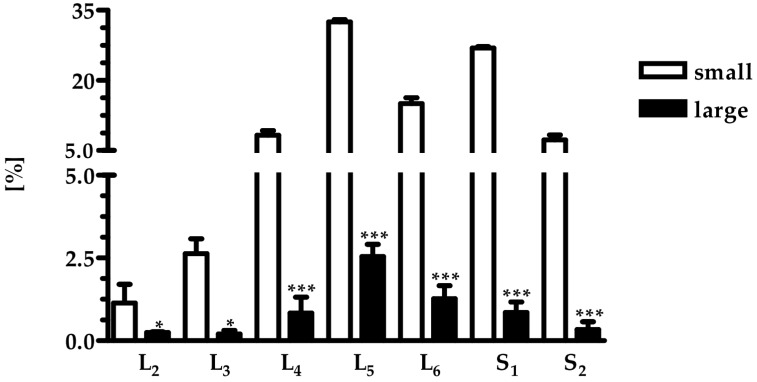
Bar diagram showing the segmental distribution of the skin-projecting neurons in porcine sympathetic chain ganglia following injection of Fast Blue in to the skin. Distribution of the skin-projecting neurons (*n* = 4). Bars represent mean number of small—(hollow bars) and large-sized (filled bars) neurons ± SEM located in left ganglia. Asterisks mark statistically significant differences: * *p* < 0.05; *** *p* < 0.001.

As revealed by the retrograde tract-tracing procedure, the SChG neurons innervating skin of the porcine hind leg were distributed in the left lumbar (L_2_–L_6_) and sacral (S_1_–S_2_) SChG (Figure 1).

**Figure 2:**
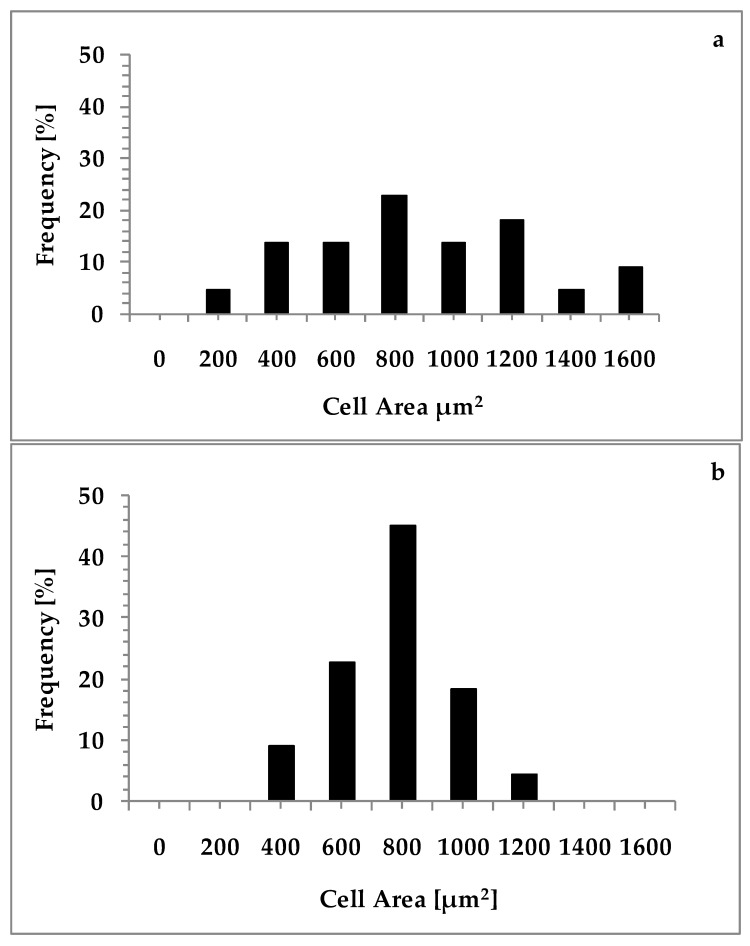
Histogram showing the relative frequency of individual subclasses of porcine skin-projecting SChG neurons, defined by their cross-sectional area in: lumbar (**a**); and sacral (**b**) SChGs (*n* = 200 neurons were measured for each histogram).

The contralateral SChG did not contain Fast Blue (FB)-labeled neurons. Among lumbar segments the highest number of these cells was observed in L_5_, while in the sacral region in S_1_. The total number of FB^+^ neurons in L_2_–S_2_ region was 3231 ± 359, and 73.7 ± 14.0% of them were located in the lumbar segments. Lumbar and sacral skin-projecting neurons were similar in size (782.1 ± 374.6 µm^2^ vs. 676.8 ± 224.6 µm^2^). Relative frequency of individual subclasses of porcine skin-projecting SChG neurons, defined by their cross-sectional area is shown in Figure 2a,b.

**Figure 3:**
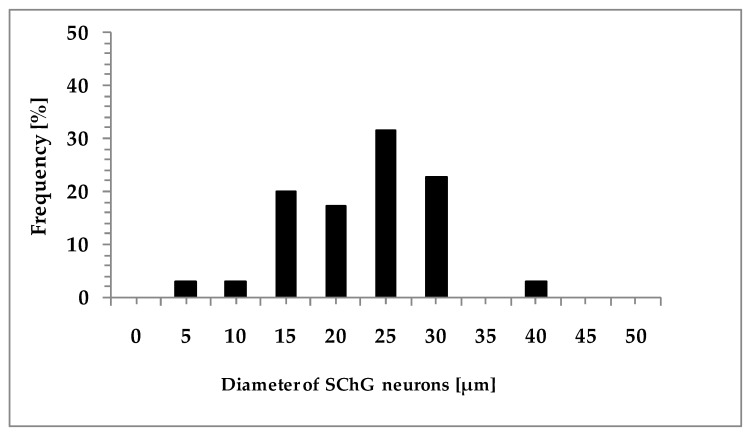
Relative frequency of cell diameter-depending classes of different the skin-projecting SChG neurons (based on *n* = 400 measured cells).

The majority of FB neurons belonged to the population of small-sized neurons, while large-sized cells was less numerous (Figure 1). Relative frequency of cell diameter-depending classes of different the skin-projecting SChG neurons is shown in Figure 3.

**Figure 4:**
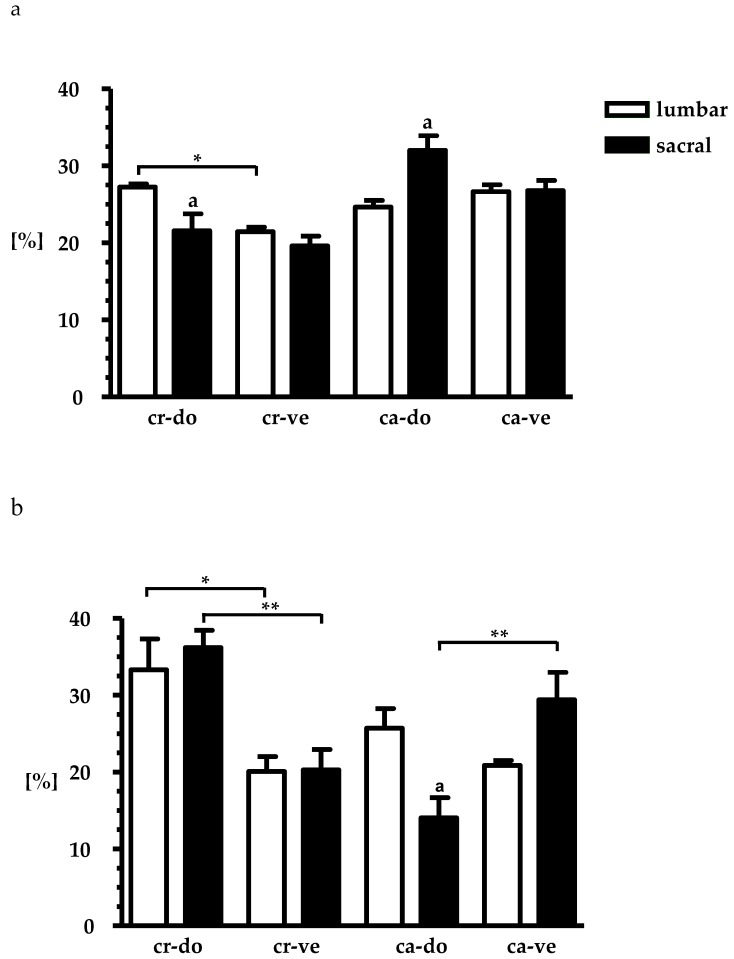
Relative frequency (mean ± SEM; *n* = 4) of skin-projecting small- and large-diameter neurons in particular subdomains of the lumbar ((**a**) L_2_–L_6_; open bars); and sacral ((**b**) S_1_–S_2_; solid bars) SChGs studied. **^a^** Indicates statistically-significant differences (*p* < 0.05) between the lumbar and sacral SChGs. * *p* < 0.05, ** *p* < 0.01 indicate statistically-significant differences between the cranio-dorsal (cr-do) and cranio-ventral (cr-ve) or caudo-dorsal (ca-do) and caudo-ventral (ca-ve) subdomains of lumbar and sacral SChGs.

When the distribution of the FB neurons was compared within four subdomains of the SChG the results were as follows (Figure 4a,b).

The small-sized cells were more often located in the cranio-dorsal subdomain (26.6 ± 0.4%) than in the cranio-ventral subdomain (21.4 ± 0.6%) of lumbar SChG. The percentage of these cells was also higher in the cranio-dorsal subdomain of lumbar SChG when compared to the sacral SChG (21.6 ± 2.2%). In the sacral SChG the highest percentage of small-sized neurons was found in the caudo-dorsal subdomain (32.0 ± 1.9%), especially when compared to the lumbar SChG (24.6 ± 0.9%). The distribution of small-sized neurons in the cranio- (22.4 ± 0.6% vs. 19.6 ± 1.3%) and caudo-ventral (26.6 ± 0.9% vs. 26.8 ± 1.3%) subdomains of the lumbar and sacral SChG was similar (Figure 4a).

The distribution patterns of the large-sized neurons within the specific subdomains of the lumbar and sacral SChG were quite different (Figure 4b). For example, large-sized neurons in the both lumbar and sacral SChG were significantly more numerous in the cranio-dorsal (33.3 ± 3.9%, 36.2 ± 2.2%; respectively) than cranio-ventral subdomains (20.0 ± 1.9%, 20.3 ± 2.6%; respectively). Moreover, in the caudo-ventral subdomain of sacral SChG, the percentage of these cells (29.4 ± 3.5%) was significantly higher when compared to the caudo-dorsal subdomain (14.0 ± 2.6%), whereas the percentage of large-sized cells localized in the latter subdomain of the lumbar SChG (25.7 ± 2.5%) was higher than in the sacral SChG (14.0 ± 2.6%). There was no statistically significant difference in the frequency of these cells between lumbar and sacral SChG in the caudo-ventral subodmain (20.8 ± 0.6% vs. 29.4 ± 3.5%). Additionally, the frequency of large-sized cells in the cranio-dorsal or cranio-ventral subdomains of the lumbar and sacral SChG was similar.

### 2.2. Neurochemical Phenotypes of Skin-Projecting Neurons within the Porcine Sympathetic China Ganglia (SChG)

All details concerning neurochemical phenotypes of skin-projecting neurons within the porcine SChG are demonstrated in Table 1 and Table 2.

**Figure 5:**
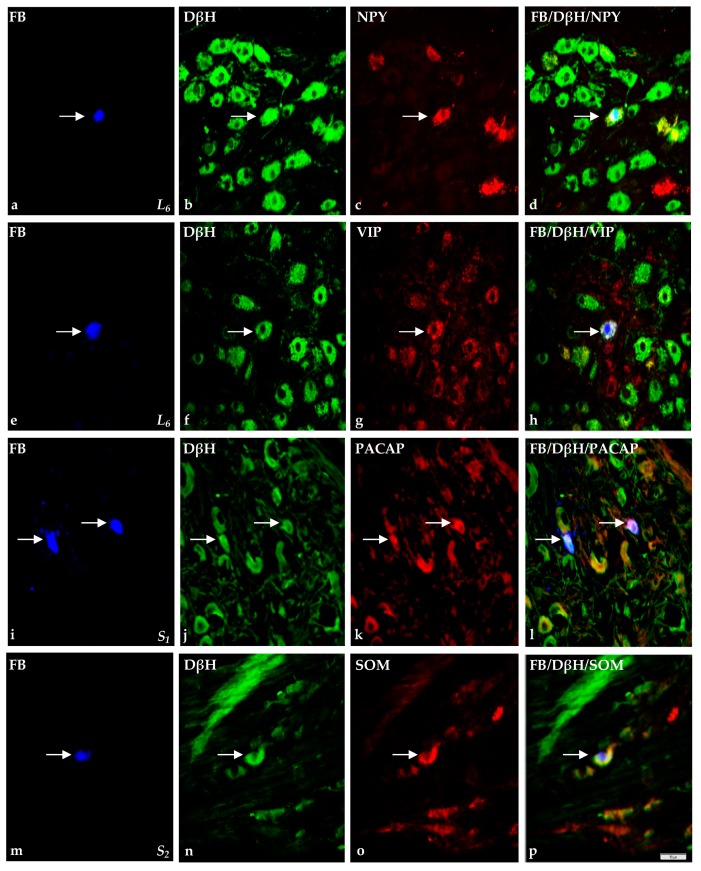
Representative images of sympathetic chain ganglia skin-projecting neurons in porcine skin hind leg. The arrow indicates Fast blue-positive (FB^+^) skin perikaryon containing examined substances. All the images are taken separately from **blue** (FB^+^ (**a**,**e**,**i**,**m**)), **green** (DβH^+^ (**b**,**f**,**j**,**n**)) and **red** (positive for: NPY (**c**); VIP (**g**); PACAP (**k**); and SOM (**o**)) fluorescent channels. Microphotographs **d**,**h**,**l**,**p** showing the over position of all three channels simultaneously. In L_6_ ganglion, FB^+^ small-sized neurons (**a**,**e**) which were simultaneously DβH^+^ (**b**,**f**) as well as NPY^+^ (**c**) and VIP^+^ (**g**). Two small-sized FB^+^ neurons (**i**) containing simultaneously DβH (**j**); and PACAP (**k**) in S_1_ SChG. Visible one FB^+^ small-sized neuron (**m**) containing DβH (**n**) and SOM (**o**) in S_2_ SChG. Scale bar = 50 µm.

**Figure 6:**
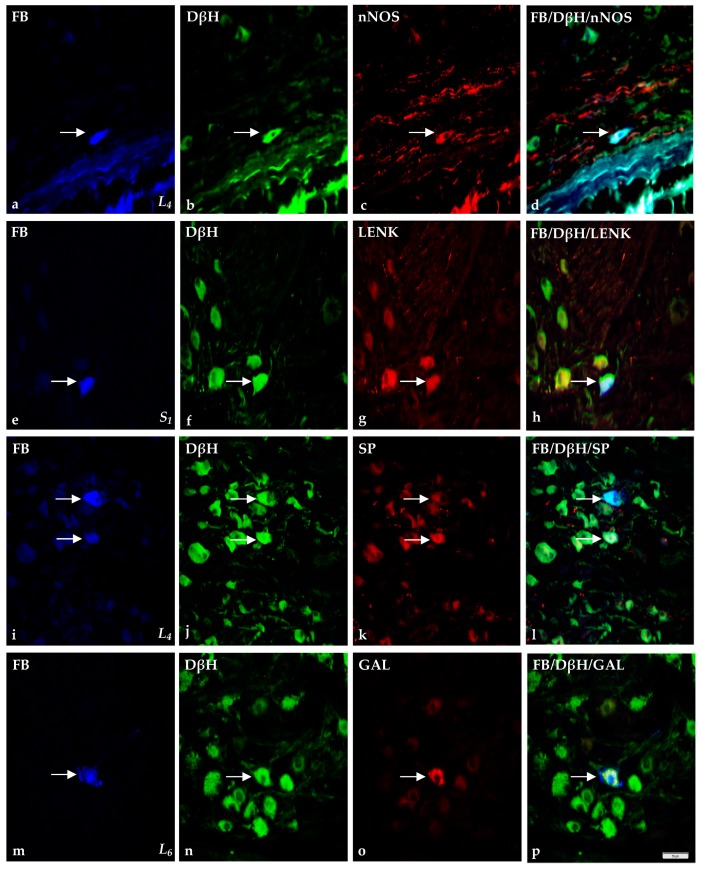
Representative images of sympathetic chain ganglia skin-projecting neurons in porcine skin hind leg. The arrow indicates Fast blue-positive (FB^+^) skin perikaryon containing examined substances. All the images are showed separately from **blue** (FB-positive (**a**,**e**,**i**,**m**)), **green** (DβH-positive (**b**,**f**,**j**,**n**)) and **red** (positive for nNOS (**c**); LENK (**g**); SP (**k**); and GAL (**o**)) fluorescent channels. Microphotographs **d**,**h**,**l**,**p** showing the over position of all three channels simultaneously. In L_4_ ganglion FB^+^ neurons (**a**,**i**) which were simultaneously DβH^+^ (**b**,**j**) as well as nNOS^+^ (**c**) and SP^+^ (**k**). Visible one FB^+^ perikaryon (**e**) containing DβH (**f**) and LENK (**g**) in S_1_ SChG. In L_6_ ganglion FB^+^ neurons (**m**) which were simultaneously DβH^+^ (**n**) as well as GAL^+^ (**o**). Scale bar = 50 µm.

The morphology and characteristic features of double-immunolabeled elements are visualized in Figure 5 and Figure 6.

Double-labeling immunohistochemistry revealed that the vast majority of the small- and large-sized skin-projecting SChG cells displayed immunoreactivity to DβH, indicating the catecholaminergic nature of these neurons. It is worth mentioning that the frequency of the small- and large-sized neurons containing this enzyme was similar in lumbar and sacral SChG (Table 3).

Moreover, these neurons also showed positivity for all other markers tested (PACAP, SOM, NOS, SP, NPY, VIP, LENK and GAL) but their percentages vary depending on the size and the location alongside the spinal cord.

#### 2.2.1. Lumbar SChG

Immunohistochemistry revealed that FB^+^/DβH^+^ small-sized cells stained for SOM, NPY and GAL were most numerous in L_4_, containing PACAP and LENK in L_5_, while the cells immunorective for FB^+^/DβH^+^/VIP^+^ (Figure 5e–h) and FB^+^/DβH^+^/SP^+^ in L_6_. In the case of large-sized neurons, FB^+^/DβH^+^/nNOS^+^ cells belonged to the numerous population in L_4_, FB^+^/DβH^+^/PACAP^+^, FB^+^/DβH^+^/VIP^+^, FB^+^/DβH^+^/NPY^+^, FB^+^/DβH^+^/LENK^+^ and FB^+^/DβH^+^/GAL^+^ in L_5_, and FB^+^/DβH^+^/SOM^+^ and FB^+^/DβH^+^/SP^+^ in L_6_. Additionally, the percentage of FB^+^/DβH^+^/SOM^+^ and FB^+^/DβH^+^/NPY^+^ small-sized neurons decreased in the caudal direction. Detailed data on the neurochemical phenotypes characteristic of skin-projecting neurons in lumbar SChG are present in Table 1.

##### L_4_ SChG

In these ganglia, the percentage of small-sized FB^+^/DβH^+^ cells stained for PACAP, SOM, SP (Figure 6i–l), VIP or NPY was significantly higher when compared to the large-sized cells. In turn, in comparison to these cells, the percentage of small-sized FB^+^/DβH^+^ cells containing nNOS was lower (Figure 6a–d). Only a few of small- and large-sized FB^+^/DβH^+^ neurons also exhibited LENK, while the latter neurons were completely devoid of GAL.

##### L_5_ SChG

In these ganglia, the percentage of FB^+^/DβH^+^ small-sized cells exhibiting nNOS was higher than large-sized neurons. Double-labeling also revealed that the percentage of the FB^+^/DβH^+^ small-sized cells that co-localized with NPY, LENK and GAL was lower than large-sized neurons containing these markers. It is worth adding that no large-sized FB^+^/DβH^+^ cells were immunoreactive for SP. It was also found that the frequency of FB^+^/DβH^+^ small- and large-sized cells exhibiting PACAP, SOM or VIP was similar in both studied populations.

##### L_6_ SChG

The percentage of FB^+^/DβH^+^ small-sized neurons also stained for nNOS, SP or VIP was higher when compared to large-sized cells. In addition, FB^+^/DβH^+^ large-sized neurons more often co-expressed SOM or NPY than small-sized neurons (NPY; Figure 5a–d). Differences in the percentages of small- and large-sized neurons containing FB^+^/DβH^+^ with PACAP, LENK or GAL (Figure 6m–p) were statistically insignificant.

#### 2.2.2. Sacral SChG

Double-labeling immunohistochemistry revealed that FB^+^/DβH^+^ small-sized cells co-expressed nNOS or GAL were most numerous in S_1_ SChG, whereas FB^+^/DβH^+^/PACAP^+^, FB^+^/DβH^+^/SOM^+^ and FB^+^/DβH^+^/VIP^+^ were most numerous in S_2_ SChG. The FB^+^/DβH^+^/SOM^+^ and FB^+^/DβH^+^/VIP^+^ large-sized neurons consisted of numerous populations in S_1_, and FB^+^/DβH^+^/PACAP^+^ and FB^+^/DβH^+^/nNOS^+^ in S_2_. Detailed data on the neurochemical phenotypes characteristic of skin-projecting neurons in sacral SChG are present in Table 2.

##### S_1_ SChG

The FB^+^/DβH^+^/SOM^+^ and FB^+^/DβH^+^/VIP^+^ small-sized neurons had lower frequency when compared to the large-sized neurons. Additionally, the frequency of FB^+^/DβH^+^/NPY^+^ small- and large-sized cells was similar. It is worth adding that the FB^+^/DβH^+^ large-sized neurons were devoid of PACAP (small-sized FB^+^ neurons; Figure 5i–l), nNOS, SP, LENK (small-sized FB^+^ neurons; Figure 6e–h) and GAL.

##### S_2_ SChG

The percentage of FB^+^/DβH^+^ small-sized neurons that co-localized with PACAP, SOM (Figure 5m–p) or VIP was higher when compared to large-sized neurons, whereas the frequency of FB^+^/DβH^+^/nNOS^+^ small-sized cells was significantly lower than that observed in large-sized neurons. No FB^+^/DβH^+^ large-sized neurons co-localized with SP, NPY, LENK and GAL were found.

## 3. Discussion

This is the first report demonstrating both the distribution and neurochemical phenotyping of sympathetic neurons projecting to skin of the porcine hindlimb. The major source of sympathetic nerve fibers supplying this region originates from the L_2_ to S_2_ SChG. These results are consistent with the data obtained from humans [21] and cats [22]. Nevertheless, in humans, these neurons were present only in lumbar SChG, while in cats they were also present in sacral ganglia.

We have also found that in the porcine skin-projecting SChG neurons may be subdivided into two size-classes: small- and large-sized neurons. The percentage of small-sized neurons was significantly higher when compared to large-sized cells in all studied SChG ganglia. These findings correlate well with results reported by Chyczewski et al. [35] and Ragionieri et al. [36], although these authors studied the population of the SChG neurons projecting to the longissimus dorsi muscle and urinary bladder in pigs, respectively. As may be judged from their size (mainly small), neurons supplying skin of the porcine hidlimb are most probably vasoconstrictors, as postulated in pig [37] and guinea pig [38].

There are other interesting observations in terms of the distribution pattern of the porcine skin-innervating neurons within subdomains of the studied ganglia, defined by ganglion geometry (i.e., the arrangement of its longest and shortest axis). It has been found that small-sized neurons were primarily found in the caudo-dorsal subdomain of sacral SChG, whereas the majority of large-sized neurons were found in the cranio-dorsal subdomain of lumbar and sacral SChG. The somatotopic distribution of sympathetic innervating different organs in pig ganglia has been observed by several authors [24,36,39,40]. Moreover, a cranio-caudal somatotopic organization, in reference to skin-projecting dorsal root ganglia neurons supplying the porcine hindlimb, has previously been postulated by Kozłowska et al. [41].

The present study has disclosed that most of the FB-labeled sympathetic small- and large-sized neurons had a catecholaminergic nature, as revealed by immunoreactivity for DβH. These results correlate well with studies describing the adrenergic character of a substantial part of the SChG neurons in the pig [23,24,25,26,27,28,37]. Only a small percentage of skin-projecting sympathetic neurons were devoid of these enzymes, which may indicate that they are a population of non-adrenergic, possibly cholinergic neurons, which is congruent with earlier studies conducted in the pig [42,43].

### 3.1. Dopamine β-Hydroxylase (DβH) and/or Neuropeptide Y (NPY)

The present data indicate that a significant amount DβH^+^ small- and large-sized perikarya also expressed NPY immunoreactivity. NPY is found in cutaneous sympathetic neurons of rodents and in humans [44,45]. Moreover, the skin-projecting neurons exhibiting DβH and NPY, in the present study, could be the predominant source of the DβH/NPY-immunorective (-IR) nerve fibers supplying skin blood vessels [46,47]. These neurons may cause vasoconstriction of these vessels, and in this way regulate blood flow through the skin [8,48,49]. Moreover, noradrenaline (NA) and NPY are unquestionably engaged in the modulation of cutaneous pain in humans [50,51,52,53]. Additionally, in the present study, the percentage of FB^+^/DβH^+^/NPY^+^ small-sized neurons in the lumbar SChG decrease from the cranial to the caudal direction. It is plausible that these neurons, distributed with different frequencies in the lumbar SChG, can modulate skin functions at different levels. Several FB^+^/NPY^+^ small- and large-sized neurons innervating the porcine skin of the hindlimb were non-adrenergic. It is possible that this infrequent population of neurons could act in dependently of NA to induce vasoconstriction, which was previously reported by Stephens et al. [30] in humans during whole body cooling. However, the exact function of non- adrenergic FB^+^/NPY^+^ skin-projecting neurons in pigs remains unexplained and requires further investigation.

### 3.2. DβH and/or Somatostatin (SOM)

The moderate number of FB^+^/DβH^+^ small- and large-sized SChG neurons was found to stain SOM. SOM has been previously found co-expressed of NA markers in neurons of the porcine SChG supplying the mammary gland [54], urinary bladder [26,27] and ovary [23]. A small proportion of FB+ small-sized neurons immunoreactive for SOM were non-adrenergic. It is worth adding that in this study the percentage of FB^+^/DβH^+^/SOM^+^ small-sized neurons in the lumbar sympathetic ganglia also decrease from the cranial to the caudal direction.

The SOM-IR nerve fibers have been found in the epidermis, dermis and, rarely, around blood vessels, sweat glands and hair follicles in human [55,56]. It should be pointed out that there is a lack of data concerning the biological function of SOM, released from nerve endings innervating skin of the hindlimb. However, it was reported that this peptide released from the stimulated sensory nerve terminals of rat sciatic nerve mediates the anti-inflammatory effect, and its receptors may play an important role in the tonic control of peripheral cutaneous nociceptors [57,58]. However, it is hard to discuss the exact biological role of FB^+^/SOM^+^/DβH^+^ SChG neurons, since the function of the SOM in connection with NA in skin has also not yet been specified.

### 3.3. DβH and/or Neuronal Nitric Oxide Synthase (nNOS)

DβH and nNOS were co-expressed in an insignificant percentage of small- and large-sized skin-projecting neurons. The presence of nNOS with DβH had earlier been observed in some SChG neurons projecting to the colon [24,25], urinary bladder [26,27] and to the extrinsic penile smooth musculature [59] of the pig. In the present study, a few small-sized non-adrenergic skin-projecting neurons (L_6_ and S_2_) were nNOS-IR. The results presented by us may explain the origin of part of the nNOS-IR terminals around microvessels and the glandular duct observed by Ibba-Manneschi et al. [60] in human skin. It is generally known that nNOS is an indicator of nitric oxide (NO), which is component of cutaneous active vasodilation in humans [61]. Whereas, the distribution of nNOS with DβH in the skin-projecting SChG neurons may implicate the involvement of endogenous NO and NA contributing to the temperature threshold of the axon reflex response to gradual local heating of skin, which was earlier observed in humans [62].

### 3.4. DβH and/or Substance (SP)

These data revealed that a moderate percentage of adrenergic skin-projecting neurons also contained SP. These co-localizations have been found in the sympathetic neurons projecting to the muscle of the porcine genital tract [59,63,64] and urinary bladder trigone [37]. Whereas DβH^+^/SP^+^ nerve fibers have been found in monkey and rat lower lip skin supplying small arteries, veins and arterioles [5]. In the present study, the presence of SP in non- adrenergic small-sized skin-projecting neurons was only observed in L_6_ SChG. This finding is similar to that observed by Botti et al. [59], where the SP-IR SChG neurons projected to one pig retractor penis muscle. In adrenergic neurons, SP probably performs an inhibitory role on the action of NA, which was previously observed in mouse spinal cord [65]. Moreover, this peptide observed by us in FB^+^ neurons with DβH and alone may partially mediate cutaneous vasodilation through NO-dependent mechanisms [66].

### 3.5. DβH and/or Vasoactive Intestinal Peptide (VIP)

The present results indicate that a part of FB^+^/DβH^+^ small- and large-sized SChG neurons has been found co-localized with VIP. This kind of co-localization was earlier observed in neurons of porcine SChG [67] projecting to the mammary gland [54], urinary bladder [26,27] and extrinsic penile smooth musculature [59]. Moreover, in the present work, VIP was also present sporadically in the non-adrenergic small-sized neurons (only in the sacral SChG) supplying skin of the porcine hindlimb, which could be a marker of cholinergic sympathetic neurons [68,69]. It was earlier reported that cutaneous VIP-IR nerve fibers supply dermal vessels, Merkel cells, hair follicles, as well as sweat, apocrine and Meibominan glands [70,71]. Based on literature data, it can be assumed that adrenergic and non-adrenergic skin-projecting neurons found in the present study may play an important role in the physiology of skin, including the regulation of blood vessels vasodilatation [72,73].

### 3.6. DβH and/or Leu5-Enkephalin (LENK)

This study showed that adrenergic small- and large-sized (only in the lumbar SChG) skin-supplying neurons sporadically co-localized with LENK. This population of neurons had already been found in porcine SChG [26,27,59]. Furthermore, in the present study, some FB^+^ small-sized neurons immunoreactive for LENK were non-adrenergic and observed only in S_1_. It seems possible that, as observed by us, adrenergic and non-adrenergic neurons are a source of very rare nerve fibers in the skin, and may decrease the release of NA and, secondarily, blood pressure, as can be seen from pharmacological studies [74,75]. The second hypothesis can be partially confirm by our data, obtained from gilts used in the current study, which showed that nerve fibers containing LENK were found in the dermis around blood vessels (Kozłowska et al., unpublished observations). However, further studies are necessary to obtain confirmation of this hypothesis.

### 3.7. DβH and/or Cyclase-Activating Polypeptide (PACAP)

DβH and PACAP were co-localized in considerable percentages within the skin-projecting SChG small- and large-sized neurons. The co-localization of these substances had already been found in the nerve fibers inside the hypothalamus of rats [76]. Moreover, in the present study some small- and large-sized FB^+^ immunoreactive for PACAP innervating porcine skin of the hindlimb were non-adrenergic. It is possible that skin-projecting SChG neurons might be the source of the DβH^+^/PACAP^+^ nerve fibers observed by Steinhoff et al. [77] close to the dermal-epidermal border, hair follicles, blood vessels and sweat glands in humans. Literature data suggest that PACAP is a vasodilatator and extravasation in the skin [78,79]. Thus, as may be judged from the above-mentioned data, PACAP with NA or alone may have a potent modulatory function in the skin’s cutaneous blood flow. However, the exact physiological role of DβH^+^/PACAP^+^ neurons in the functioning of skin of the hindlimb is still unknown.

### 3.8. DβH and/or GAL

This study revealed that FB^+^/DβH^+^/GAL^+^ small- and large-sized neurons represent a portion of the retrogradely labeled cells. Additionally, the population of skin-projecting neurons was completely devoid of non-adrenergic GAL-IR cells. The co-existence of these substances was previously observed in SChG supplying the ovary [23]. The immunohistochemical study in humans demonstrated that GAL-IR nerve fibers in the skin were distributed around eccrine sweat glands, apocrine glands and blood vessels [80,81]. It was also reported that receptor 3 for the GAL was associated with dermal blood vessels, which suggests a role for this peptide in the regulation of microvasculature in skin [82]. This suggestion can be confirmed via a reduction of cutaneous plasma extravasationin GAL-overexpressing mice after activation of neurogenic inflammation [83]. Additionally, GAL may participate in nociceptive transmission, which was noted in mice [84].

In conclusion, the present study provides for the first time a detailed description of both the source of sympathetic nerve fibers innervating the skin of the porcine hindlimb and chemical phenotyping of neurons being the source of these fibers. Moreover, the differences observed by us between the studied ganglia in the percentage of DβH-IR neurons co-localized with NPY, PACAP, VIP, SOM, nNOS, LENK, SP or GAL, suggest that these cells may play a role of modulators of sympathetic control at varying levels of skin function in pigs. Further research should be carried out to elucidate in detail the physiological relevance of each subpopulation of neurons innervating skin of the porcine hindlimb.

## 4. Materials and Methods

### 4.1. Laboratory Animals

The experiment was performed on 4 juvenile female crossbred gilts (Pietrain × Duroc), aged 8–12 weeks and weighting 15–20 kg. All animals were housed and treated in accordance with the Principles of Laboratory Animal Care (NIH publication no. 86-23, revised 1985). All experimental procedures were approved by the Local Ethical Commission of the University of Warmia and Mazury in Olsztyn (No. 36/2013). Seven days before surgical operations the animals were transported from a farm to the local animal house, where they were individually housed in stalls, under conditions of natural light and room temperature. All animals were fed with a commercial grain mixture (Grower Plus, Wipasz, Wadąg, Poland), and they had free access to water. 24 h before surgery feeding was stopped. All efforts were made to minimize the number of animals used and their suffering.

### 4.2. Anesthesia, Surgery and Tissue Processing

Thirty minutes before the main anesthetic was given, the animals were pretreated with atropine sulfate (Polfa, Warsaw, Poland; 0.04 mg/kg b.w., s.c.) and azaperone (Stressnil, Janssen Pharmaceutica, NV, Beerse, Belgium; 0.5 mg/kg b.w., i.m). All animals were anesthetized with sodium thiobarbital (Thiopenthal, Sandoz, Holzkirchen, Germany; 20 mg/kg b.w., i.v., inafractionate dinfusion). After induction of the surgical anesthesia, the skin of the left hindlimb was gently shaved, disinfected with 1% water-alcoholic solution of iodinetincture, and then a total volume of 100 μL of 5% aqueous solution of the fluorescent retrograde tracer Fast Blue (FB; EMS-Chemie GmbH, Groß-Umstadt, Hesse, Germany) was administered in multiple injections in to the external surface of the skin using a Hamilton microsyringe equipped with a 26S gauge needle. Injections were made at equal intervals of one centimeter, and they strictly covered the skin area innervated by cutaneous branches of the femoral and sciatic nerves (100 μL) [41]. Four weeks later all the animals were euthanized by an overdose of sodium thiobarbital and, after cessation of breathing and heart beating, transcardially perfused with 4% buffered paraformaldehyde (pH 7.4). Bilateral SChG (Th_12_–S_4_) were collected from all animals studied and then postfixed by immersion in the same fixative for 15 min, washed twice in 0.1 M phosphate buffer (pH 7.4, 4 °C) for over three days, and then stored in 18% buffered sucrose solution containing 0.01% sodium azide (pH 7.4) at 4 °C until they sank. Finally, the tissues were frozen and then coronally sectioned at a thickness of 10μm using a cryostat (HM525 Zeiss, Jena, Germany). The sections were stored at −80 °C until further processing.

### 4.3. Immunofluorescence Procedures

The sections were processed for routine double-immunofluorescence labeling using primary antisera, raised in different species and species-specific secondary antibodies listed in Table 4.

Immunohistochemistry involved double-staining, which was applied to sections from L_4_–L_6_, S_1_ and S_2_ ganglia. The sections were selected from three different representative regions of the ganglia (upperone-third, middle and lower one-third).

Due to the presence of FB+ neurons in the studied sections, all steps of the procedures described below were performed in darkness. Thus, sections were air-dried at room temperature (RT) for 45 min, rinsed three times in phosphate-buffered saline (PBS, pH 7.4), and then, in order to block all the unspecific binding sites, incubated for 1h in a humid chamber with a blocking solution containing 1% Triton X-100 (Sigma-Aldrich, St. Louis, MO, USA), 0.1% bovine serum albumin (Sigma-Aldrich, St. Louis, MO, USA), 0.05% thimerosal (Sigma-Aldrich, St. Louis, MO, USA), 0.01% NaN_3_ (POCH, Gliwice, Poland) and 10% normal goat serum (Jackson Immunoresearch, West Grove, PA, USA) in 0.01 M PBS.

For the double-labeling immunofluorescence, a mixture of two primary antibodies was used. After the incubation with primary antibodies, sections were rinsed in PBS (3 × 15 min) and incubated for 1 h with a mixture of appropriate FITC-conjugated secondary antisera and biotinylated donkey anti-rabbit antibodies. The latter antibodies were finally visualized by additional incubation of sections with streptavidin-CY3 complex for 1h. Following subsequent rinsing in PBS (3 × 15 min), the sections were coverslipped with carbonate-buffered glycerol (pH 8.6).

### 4.4. Counting of Neurons and Statistical Analysis

The sections were viewed under an Olympus BX61 microscope (Shinjuku, Tokyo, Japan) equipped with epi-fluorescence and appropriate, small-band filter sets for FB, FITC and CY3. Images were acquired by a PC equipped with a CCD camera operated by CellSens Dimension image analyzing software (Olympus, Warszawa, Poland).

**Figure 7:**
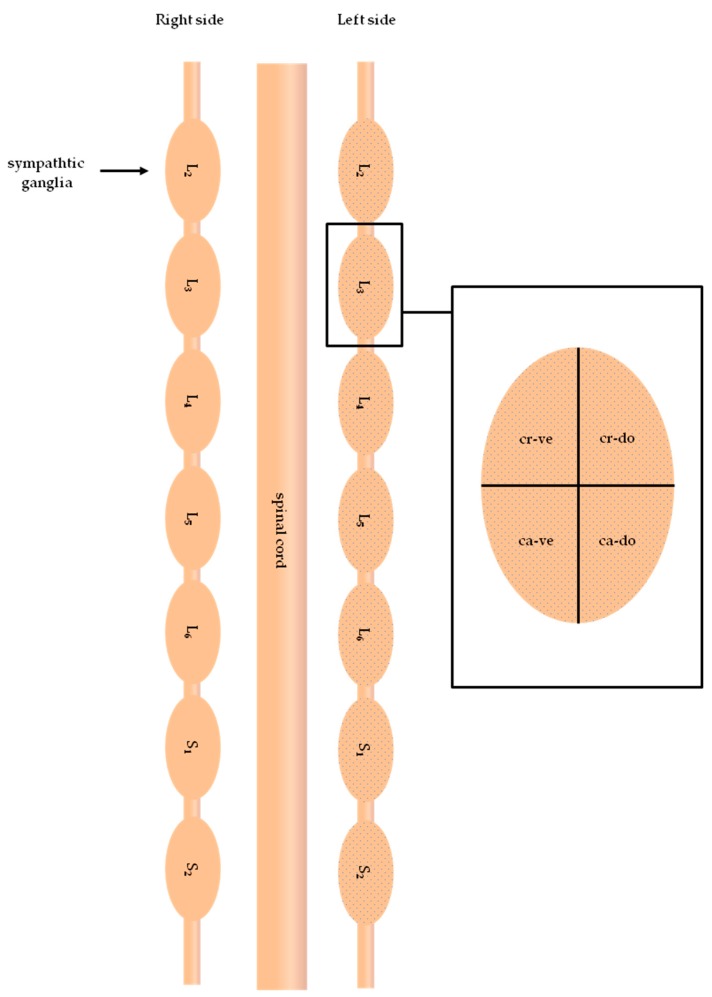
A schematic diagram of sympathetic chain ganglia (SChG) showing topographical subdomains in which the relative frequencies of skin projecting neurons were counted. cr-do, cranio-dorsal; cr-ve, cranio-ventral; ca-do, caudo-dorsal; and ca-ve, caudo-ventral subdomains of the SChG.

To avoid a double-counting of labeled neurons, only FB-positive perikary a with a clearly visible nucleus were counted in each fourth section of the particular SChG studied. Moreover, to further unravel the intraganglionic distribution of FB-traced neurons, each of the SChG sections of interest was divided, along their long and short axes, into four subdomains: cranio-dorsal, cranio-ventral, caudo-dorsal and caudo-ventral (Figure 7).

**Figure 8:**
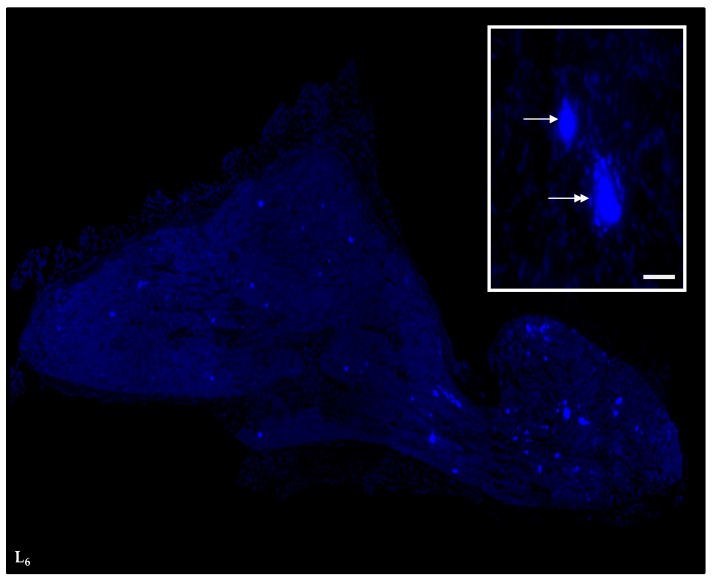
Consecutive microphotographs-based reconstruction of a paramedian section of the L_6_ SChG demonstrating intraganglionic distribution pattern of retrogradely-labeled small- (<25 µm) and large-sized (>25 µm) skin-projecting neurons. Insert: a fragment of a microphotograph made at a “working” magnification used for morphometric analysis of retrogradely-labeled perikarya; a typical small-sized neuron is indicated by an arrow, while a double-headed arrow points out to a large-sized cell. Scale bar in insert = 50 µm.

The perikarya were divided into two subclasses according to diameter: small (<25 µm in diameter; mean 17.4 ± 6.0) and large (>25 µm in diameter; mean 29.6 ± 5.4; Figure 8). Their size was measured using CellSens Dimension image analyzing software.

FB+ neurons were counted with in individual lumbar and sacral SChG, as well as within each of the ganglionic subdomains. These counts were expressed as the percentages of small- and large-sized FB^+^ neurons in each of the SChG or ganglionic subdomains, always considering the total number of FB^+^ neurons as 100% within each the studied segment or ganglionic subdomains. A similar paradigm was used to evaluate the inter- as well as intra-ganglionic distribution patterns of the double-immunolabeled neurons. However, the relative frequencies of neurons within ganglionic subdomains were calculated from the pooled values of FB-labeled neurons in lumbar SChG (L_4_–L_6_) and sacral SChG (S_1_ and S_2_).

Data concerning the segmental distribution and the number of skin-projecting sympathetic perikarya in the total population of lumbar and sacral SChG were analyzed using the Mann–Whitney *U*-test. The statistical importance of differences found between ganglia of data was analyzed using one-way ANOVA followed by the Tukey test using GraphPad Prism4 software (GraphPad Software, LaJolla, CA, USA). Differences between population small- and large-sized neurons innervating skin of the hindlimb were compared using the Mann–Whitney *U*-test. *p* < 0.05 was considered to be statistically significant.

### 4.5. Specificity Tests of Tracing and Labeling Procedures

After a careful examination of each injection site, traces of FB were found neither in muscles, nor in the subcutaneous tissues in the vicinity of the tracer deposition places. The specificity of primary or secondary antisera was tested by: (1) preabsorption tests based on incubation with an antibody that had been preabsorbed with synthetic antigen (25 μg of appropriate antigen per 1 mL of corresponding antibody at working dilution); and (2) omission and replacement tests during which the primary or secondary antibody was omitted or replaced by non-immune sera or PBS. Lack of any visible fluorescence indicated the specificity of the labeling.

## 5. Conclusions

In conclusion, the present study provides for the first time a detailed description of both the source of sympathetic nerve fibers innervating the skin of the porcine hindlimb and chemical phenotyping of neurons being the source of these fibers. Moreover, the differences observed by us between the studied ganglia in the percentage of DβH-IR neurons co-localized with NPY, PACAP, VIP, SOM, nNOS, LENK, SP or GAL suggest that these cells may play a role of modulators of sympathetic control at varying levels of skin function in pigs. Further research should be carried out to elucidate in detail the physiological relevance of each subpopulation of neurons innervating skin of the porcine hindlimb.

## Figures and Tables

**Table 1:** Percentages of retrogradely labeled small- and large-sized neurons in the porcine lumbar (L_4_–L_6_) sympathetic chine ganglia projecting to the skin of the hindlimb.

Substances	Ganglia											
	L_4_				L_5_				L_6_			
	FB^+^/DβH^+^/S^+^	FB^+^/DβH^+^/S^−^	FB^+^/DβH^−^/S^+^	FB^+^/DβH^−^/S^−^	FB^+^/DβH^+^/S^+^	FB^+^/DβH^+^/S^−^	FB^+^/DβH^−^/S^+^	FB^+^/DβH^−^/S^−^	FB^+^/DβH^+^/S^+^	FB^+^/DβH^+^/S^−^	FB^+^/DβH^−^/S^+^	FB^+^/DβH^−^/S^−^
PACAP	Small cells				Small cells				Small cells			
	26.0 ± 1.3	49.7 ± 1.8	0.3 ± 0.3	23.8 ± 1.3	49.5 ± 2.3 ^a (*p* < 0.001)^	34.8 ± 4.6 ^a (*p* < 0.05)^	0.6 ± 0.6	14.9 ± 4.0	33.1 ± 1.9 ^c (*p* < 0.001)^	39.6 ± 3.4	0	27.3 ± 1.9 ^c (*p* < 0.01)^
	Large cells				Large cells				Large cells			
	18.1 ± 1.7 *	61.4 ± 1.7 ***	0	20.5 ± 0.5	36.1 ± 7.3	57.6 ± 4.8	0	6.2 ± 6.2	20.9 ± 0.5	58.1 ± 1.0	0	20.9 ± 0.5
SOM	Small cells				Small cells				Small cells			
	33.7 ± 2.2	52.8 ± 2.9	1.0 ± 0.6	12.4 ± 1.9	23.5 ± 0.7 ^a (*p* < 0.001)^	62.6 ± 1.3	0.3 ± 0.3	13.6 ± 1.2	17.4 ± 0.9 ^b(*p*<0.001);c(*p*<0.001)^	62.9 ± 3.5	2.7 ± 1.7	16.9 ± 1.4
	Large cells				Large cells				Large cells			
	23.9 ± 3.2 **	63.3 ± 1.9*	0	12.8 ± 4.7	25.1 ± 0.02	43.9 ± 2.0 ***^; a (*p* < 0.001)^	0	31.0 ± 2.0 ***^; a (*p* < 0.001)^	34.1 ± 1.3 ***^; b (*p* < 0.01); c (*p* < 0.05)^	50.3 ± 0.6 **^, b (*p* < 0.01)^	0	15.6 ± 0.7 ^c (*p* < 0.01)^
nNOS	Small cells				Small cells				Small cells			
	11.2 ± 0.6	68.8 ± 2.7	0	20.0 ± 2.6	15.9 ± 0.9 ^a (*p* < 0.05)^	73.6 ± 0.8	0	10.4 ± 0.6 ^a (*p* < 0.02)^	15.8 ± 0.4 ^b (*p* < 0.05)^	68.9 ± 1.1	0.7 ± 0.4	14.5 ± 1.8
	Large cells				Large cells				Large cells			
	17.9 ± 1.9 **	62.8 ± 1.5	0	19.2 ± 1.0	11.4 ± 0.7 *^, a (*p* < 0.01)^	53.6 ± 1.3 ***^, a (*p* < 0.01)^	0	35.0 ± 1.6 ***^, a (*p* < 0.001)^	11.7 ± 0.5 ***	44.2 ± 0.7 ***^; b (*p* < 0.001); c (*p* < 0.01)^	0	44.1 ± 0.8 ***^; b (*p* < 0.001); c (*p* < 0.01)^
SP	Small cells				Small cells				Small cells			
	21.9 ± 0.5	59.4 ± 0.8	0	18.7 ± 0.6	22.2 ± 0.9	57.7 ± 0.4	0	19.9 ± 0.5	35.1 ± 0.4 ^b ( *p* < 0.001)^	56.3 ± 1.0	0.5 ± 0.5	8.1 ± 0.9 ^b (*p* < 0.001); c (*p* < 0.001)^
	Large cells				Large cells				Large cells			
	7.8 ± 0.3 ***	70.8 ± 0.6 *	0	21.4 ± 0.5 *	0	56.2 ± 5.5 ^a ( *p*< 0.01)^	0	43.7 ± 5.5 ***^; a(*p* < 0.001)^	12.1 ± 0.3 ***^; b (*p* < 0.001)^	54.5 ± 0.7 ^b (*p* < 0.01)^	0	33.3 ± 0.4 ***^; b (*p* < 0.05); c (*p* < 0.05)^
VIP	Small cells				Small cells				Small cells			
	19.5 ± 1.1	70.0 ± 1.2	0	10.4 ± 0.4	20.0 ± 0.8	73.5 ± 0.4	0	6.5 ± 0.4	25.5 ± 1.5 ^b (*p* < 0.01); c (*p* < 0.05)^	64.9 ± 1.0 ^c (*p* < 0.01)^	0	9.6 ± 0.5
	Large cells				Large cells				Large cells			
	3.6 ± 1.1 ***	94.6 ± 2.1 **	0	1.8 ± 1.8 ***	21.7 ± 0.6 ^a (*p* < 0.001)^	33.9 ± 3.2 ***^, a (*p* < 0.001)^	0	44.4 ± 1.1 ***^, a (*p* < 0.001)^	9.2 ± 0.8 ***^, b (*p* < 0.01); c (*p* < 0.001)^	60.3 ± 0.8 ^b (*p* < 0.001); c (*p* < 0.001)^	0	30.5 ± 1.0 ***^; b (*p* < 0.001); c (*p* < 0.001)^
NPY	Small cells				Small cells				Small cells			
	37.6 ± 0.5	59.1 ± 1.4	0.2 ± 0.2	3.1 ± 0.7	26.9 ± 1.0 ^a (*p* < 0.01)^	68.9 ± 1.2	1.0 ± 1.0	2.7 ± 2.7	18.3 ± 0.2 ^b (*p* < 0.001); c (*p* < 0.05)^	70.8 ± 1.3 ^b (*p* < 0.001)^	0	10.8 ± 1.1 ^b (*p* < 0.05); c (*p* < 0.05)^
	Large cells				Large cells				Large cells			
	21.8 ± 0.8 ***	58.2 ± 0.9	2.7 ± 2.7	17.2 ± 2.2 ***	55.8 ± 3.9 ***^, a (*p* < 0.001)^	30.2 ± 1.1 ***^; a (*p* < 0.001)^	2.7 ± 2.7	11.2 ± 0.7*	32.9 ± 1.4 ***^; b (*p* < 0.001); c (*p*<0.001)^	51.5 ± 0.8 ***^; c (*p* < 0.001)^	0	15.4 ± 0.9
LENK	Small cells				Small cells				Small cells			
	1.3 ± 0.6	88.8 ± 1.5	0	9.9 ± 0.8	3.4 ± 0.6	82.2 ± 1.2	0	14.1 ± 1.0 ^a (*p* < 0.05)^	1.8 ± 0.9	85.8 ± 2.4	0	12.4 ± 0.4
	Large cells				Large cells				Large cells			
	1.6 ± 1.0	78.4 ± 3.7 *	0	20.0 ± 0.6 ***	11.4 ± 0.7 ***^; a (*p* < 0.001)^	77.1 ± 0.4	0	11.4 ± 0.7 ^a (*p* < 0.001)^	0.5 ± 0.5 ^c (*p* < 0.001)^	87.8 ± 1.5	0	11.5 ± 1.2
GAL	Small cells				Small cells				Small cells			
	26.6 ± 3.6	63.0 ± 3.7	0	10.3 ± 1.3	9.0 ± 0.2 ^a ( *p* <0.001)^	80.7 ± 2.2 ^a ( *p* <0.001)^	0	10.0 ± 0.4	14.3 ± 0.2 ^b (*p* < 0.001)^	71.0 ± 0.4 ^b (*p* < 0.05); c (*p* < 0.05)^	0	14.5 ± 0.5 ^b (*p* < 0.05)^
	Large cells				Large cells				Large cells			
	0	85.1 ± 1.1 ***	0	14.8 ± 1.1 *	13.5 ± 0.6	73.6 ± 0.9 ^a (*p* < 0.01)^	0	12.8 ± 0.4	11.5 ± 0.2^)^	73.9 ± 0.9 ^b (*p* < 0.01)^	0	14.5 ± 0.9

Data were expressed as mean ± standard error (± SEM). S—studied substance (NPY, SOM, VIP, etc.). *, **, and *** indicate statistically-significant differences (*p* < 0.05, *p* < 0.01, and *p* < 0.001, respectively) between the small- and large-sized neurons in the SChGs. ^a^ Indicates statistically-significant differences (*p* < 0.05, *p* < 0.01, and *p* < 0.001) between the L_4_ and L_5_ segments of the SChG. ^b^ Indicates statistically-significant differences (*p* < 0.05, *p* < 0.01, and *p* < 0.001) between the L_4_ and L_6_ segments of the SChG. ^c^ indicates statistically-significant differences (*p* < 0.05, *p* < 0.01, *p* < 0.001) between the L_5_ and L_6_ segments of the SChG. The total number of cells counted was 6791. The mean number of cells counted for each combination was 289 ± 16.2.

**Table 2:** Percentages of retrogradely labeled small and large cells in the porcine sacral (S_1_–S_2_) sympathetic chine ganglia projecting to the skin of the hindlimb.

Substances	Ganglia							
	S_1_				S_2_			
	FB^+^/DβH^+^/S^+^	FB^+^/DβH^+^/S^−^	FB^+^/DβH^−^/S^+^	FB^+^/DβH^−^/S^−^	FB^+^/DβH^+^/S^+^	FB^+^/DβH^+^/S^−^	FB^+^/DβH^−^/S^+^	FB^+^/DβH^−^/S^−^
PACAP	Small cells				Small cells			
	34.4 ± 0.8	59.4 ± 1.6	0	6.2 ± 0.7	52.5 ± 1.2 ^a (*p* < 0.001)^	39.1 ± 0.5 ^a (*p* < 0.001)^	2.9 ± 1.6	5.4 ± 0.6
	Large cells				Large cells			
	0	62.2 ± 1.0 ***	0	37.7 ± 1.0 ***	31.1 ± 0.9 ***	54.2 ± 0.9 ***^; a (*p* < 0.001)^	0	14.7 ± 0.3 ***^; a (*p* < 0.001)^
SOM	Small cells				Small cells			
	32.1 ± 5.2	47.8 ± 2.2	0	19.9 ± 4.5	41.8 ± 0.6 ^a (*p* < 0.05)^	42.3 ± 0.7	2.2 ± 0.9	13.7 ± 0.7
	Large cells				Large cells			
	57.1 ± 0.4 ***	26.9 ± 0.7 ***	0	15.9 ± 0.5	25.4 ± 0.7 ***^; a (*p* < 0.001)^	55.3 ± 0.9 ***^; a (*p* < 0.01)^	0	19.2 ± 0.2
nNOS	Small cells				Small cells			
	15.1 ± 0.7	75.0 ± 0.5	0	9.9 ± 0.3	4.5 ± 0.05 ^a (*p* < 0.001)^	76.4 ± 0.4	2.2 ± 0.4	16.9 ± 0.7 ^a (*p* < 0.001)^
	Large cells				Large cells			
	0	100	0	0	49.8 ± 0.1 ***	50.2 ± 0.1 ***	0	0
SP	Small cells				Small cells			
	19.0 ± 1.3	66.8 ± 0.9	0	14.1 ± 0.9	20.8 ± 0.1	65.0 ± 1.6	0	14.2 ± 1.1
	Large cells				Large cells			
	0	100	0	0	0	76.7 ± 1.1 ***	0	23.2 ± 1.1 ***
VIP	Small cells				Small cells			
	17.7 ± 0.5	64.7 ± 3.4	1.1 ± 1.1	16.5 ± 1.3	28.2 ± 0.7 ^a ( *p*< 0.05)^	57.8 ± 3.3	0.9 ± 0.9	13.1 ± 1.7
	Large cells				Large cells			
	35.2 ± 0.8 ***	50.6 ± 0.7 ***	0	14.2 ± 0.8	13.6 ± 1.1 **^; a (*p* < 0.001)^	86.3 ± 1.1 ***^; a (*p* < 0.001)^	0	0
NPY	Small cells				Small cells			
	25.9 ± 1.5	62.7 ± 3.0	0	11.3 ± 3.9	27.3 ± 3.5	60.2 ± 4.5	0.3 ± 0.3	12.0 ± 1.1
	Large cells				Large cells			
	33.3 ± 1.1	55.9 ± 0.5	0	10.7 ± 0.7	0	50.6 ± 0.6	0	49.3 ± 0.6 ***^; a (*p* < 0.001)^
LENK	Small cells				Small cells			
	5.7 ± 2.6	67.5 ± 1.3	0.6 ± 0.6	25.9 ± 1.5	2.8 ± 1.4	74.4 ± 1.4	0	22.7 ± 1.6
	Large cells				Large cells			
	0	52.5 ± 1.6 ***	0	47.5 ± 3.1 ***	0	79.0 ± 1.3 ^a (*p* < 0.001)^	0	21.0 ± 1.2 ^a (*p* < 0.001)^
GAL	Small cells				Small cells			
	23.0 ± 0.2	61.6 ± 0.9	0	15.3 ± 0.8	12.7 ± 0.9 ^a (*p* < 0.001)^	67.6 ± 1.2 ^a (*p* < 0.01)^	0	19.7 ± 1.0 ^a (*p* < 0.001)^
	Large cells				Large cells			
	0	100	0	0	0	85.0 ± 1.0 ***	0	15.0 ± 1.0 *

Data were expressed as mean ± standard error (±SEM). S—studied substance (NPY, SOM, VIP, etc.). *, **, and *** indicate statistically-significant differences (*p* < 0.05, *p* < 0.01, and *p* < 0.001, respectively) between the small- and large-sized neurons in the SChGs. ^a^ Indicates statistically-significant differences (*p* < 0.05, *p* < 0.01, and *p* < 0.001) between the S_1_ and S_2_ segments of the SChG. The total number of cells counted was 2670. The mean number of cells counted for each combination was 166 ± 58.1.

**Table 3:** Percentages of retrogradely labeled small- and large-sized neurons in the porcine lumbar (L_4_–L_6_) and sacral (S_1_–S_2_) sympathetic chine ganglia projecting to the skin of the hindlimb, which were simultaneously dopamine β-hydroxylase-positive (DβH^+^) or -negative (DβH^−^).

Size of the FB^+^ Neurons	Marker	L_4_	L_5_	L_6_	S_1_	S_2_
small	DβH^+^	86.1 ± 6.6	87.4 ± 4.3	85.0 ± 6.2	83.1 ± 5.4	84.0 ± 4.5
	DβH^−^	13.8 ± 6.6	12.5 ± 4.3	14.9 ± 6.2	16.8 ± 5.4	15.9 ± 4.5
large	DβH^+^	84.3 ± 6.9	75.1 ± 15.5	77.0 ± 11.1	84.2 ± 17.9	82.1 ± 15.5
	DβH^−^	15.6 ± 6.9	24.8 ± 15.5	22.9 ± 11.1	15.7 ± 17.9	17.8 ± 15.5

Data were expressed as mean ± standard error (±SEM).

**Table 4:** Primary antisera, secondary reagents and dilutions used in the study (DβH, dopamine-β-hydroxylase; NPY, neuropeptide Y; VIP, vasoactive intestinal polypeptide; PACAP, pituitary adenylate cyclase-activating polypeptide, SOM, somatostatin; nNOS, neuronal nitric oxide synthase; LENK, leu5-enkephalin; SP, substance P; GAL, galanin; and FITC, fluorescein isothiocyanate).

**Primaryantisera**				
**Antigen**	**AntibodyCode**	**HostSpecies**	**Dilution**	**Supplier**
DβH	MAB308	Mouse	1:1000	Merck Millipore; Billerica, MA, USA
NPY	NA1233	Rabbit	1:8000	Enzo Life Sciences; Farmingdale, NY, USA
VIP	VA1285	Rabbit	1:4000	Enzo Life Sciences; Farmingdale, NY, USA
PACAP	H-052-02	Rabbit	1:11,000	Phoenix Pharmaceuticals, Inc., Belmont, CA
SOM	AB5494	Rabbit	1:4000	Merck Millipore, Billerica, MA, USA
nNOS	AB5380	Rabbit	1:13,000	Merck Millipore, Billerica, MA, USA
LENK	EA1149	Rabbit	1:9000	Enzo Life Sciences; Farmingdale, NY, USA
SP	8450-0004	Rabbit	1:3000	Bio-Rad (formerly AbD Serotec); Langford, UK
GAL	AB5909	Rabbit	1:16,400	Merck Millipore; Billerica, MA, USA
**Secondary Reagents**				
**Reagent**	**Conjugated to**	**Antibody Code**	**Dilution**	**Supplier**
Donkey anti-mouse IgG(H+L)	FITC	715-095-151	1:1000	Jackson I.R. Inc. West Baltimore Pike, West Grove, PA, USA
Biotinylated goat anti-rabbit immunoglobulins	biotin	E0432	1:1500	Dako; ‎Glostrup, Germany
Streptavidin	CY3	016-160-084	1:10,000	Jackson I.R. Inc. West Baltimore Pike, West Grove, PA, USA
